# Supplementary material for: Camel milk is a neglected source of brucellosis among rural Arab communities
Source: Nat Commun. 2025 Jan 20;16:861. doi: 10.1038/s41467-024-55737-2 (PMC11756418; doi:10.1038/s41467-024-55737-2)
Supplement: Supplementary file 2 — Reporting Summary [file 41467_2024_55737_MOESM2_ESM.pdf]

nature portfolio

Corresponding author(s): Peter Holloway

Last updated by author(s): Dec 7, 2024

Reporting Summary

Nature Portfolio wishes to improve the reproducibility of the work that we publish. This form provides structure for consistency and transparency in reporting. For further information on Nature Portfolio policies, see our Editorial Policies and the Editorial Policy Checklist.

Statistics

For all statistical analyses, confirm that the following items are present in the figure legend, table legend, main text, or Methods section.

n/a	Confirmed	
☐	☒	The exact sample size (*n*) for each experimental group/condition, given as a discrete number and unit of measurement
☒	☐	A statement on whether measurements were taken from distinct samples or whether the same sample was measured repeatedly
☐	☒	The statistical test(s) used AND whether they are one- or two-sided<br>*Only common tests should be described solely by name; describe more complex techniques in the Methods section.*
☐	☒	A description of all covariates tested
☐	☒	A description of any assumptions or corrections, such as tests of normality and adjustment for multiple comparisons
☐	☒	A full description of the statistical parameters including central tendency (e.g. means) or other basic estimates (e.g. regression coefficient) AND variation (e.g. standard deviation) or associated estimates of uncertainty (e.g. confidence intervals)
☐	☒	For null hypothesis testing, the test statistic (e.g. *F*, *t*, *r*) with confidence intervals, effect sizes, degrees of freedom and *P* value noted<br>*Give P values as exact values whenever suitable.*
☐	☒	For Bayesian analysis, information on the choice of priors and Markov chain Monte Carlo settings
☐	☒	For hierarchical and complex designs, identification of the appropriate level for tests and full reporting of outcomes
☐	☒	Estimates of effect sizes (e.g. Cohen's *d*, Pearson's *r*), indicating how they were calculated

Our web collection on statistics for biologists contains articles on many of the points above.

Software and code

Policy information about availability of computer code

Data collection	Open Data Kit (version 1.10) .
Data analysis	R (version 4.0.3)

For manuscripts utilizing custom algorithms or software that are central to the research but not yet described in published literature, software must be made available to editors and reviewers. We strongly encourage code deposition in a community repository (e.g. GitHub). See the Nature Portfolio guidelines for submitting code & software for further information.

Data

Policy information about availability of data

All manuscripts must include a data availability statement. This statement should provide the following information, where applicable:

- Accession codes, unique identifiers, or web links for publicly available datasets
- A description of any restrictions on data availability
- For clinical datasets or third party data, please ensure that the statement adheres to our policy

Our study involves human subjects in a sparsely populated area, where the combination of variables in the dataset could potentially identify individuals even without direct identifiers such as names or exact geolocations. We will make the data, or a subset, available upon reasonable requests accompanied by a proposal aligned with the ethical approval granted for the study and potentially a data access agreement to ensure compliance with ethical and legal regulations. The data availability statement in the manuscript is as follows, 'All authors had full access to all data in the study. The data supporting the findings of this study, along with study questionnaires, is available upon reasonable request to the corresponding author, subject to approval by the relevant ethics committees'.

Research involving human participants, their data, or biological material

Policy information about studies with human participants or human data. See also policy information about sex, gender (identity/presentation), and sexual orientation and race, ethnicity and racism.

Reporting on sex and gender	Due to cultural sensitivity, information regarding participant sex was obtained from interviewer observation, and included as an a priori variable in logistic analysis analyses.
Reporting on race, ethnicity, or other socially relevant groupings	Information regarding nationality was self-reported, according to national identity card or passport details.
Population characteristics	Study participants were members of livestock owning households in southern Jordan, included in Ministry of Agriculuture compiled lists of livestock owners in Aqaba and Ma'an governorates.
Recruitment	Recruitment of all human research participants was performed in accordance with the Declaration of Helsinki, including informed consent, with a small gift (a blanket, toothbrush, toothpaste & soap) given as compensation for participation. We conducted a cross-sectional study between September 27th, 2017, and October 11th, 2018, among livestock-owning households in four sub-regions of southern Jordan (Aqaba East, Aqaba West, Ma'an East, and Ma'an West) (Figure 3). Livestock-owning households were randomly selected by a co-author (SN) using computer-generated randomisation lists (Stata, version 15.1) from a sampling frame provided by the MoA, comprising a list of all households owning one or more camels, and a list of those owning sheep or goats, within the four sub-regions. Both house and tent dwellings were eligible for inclusion. All household members present at the time of sampling were invited to participate, except children younger than 5 years or individuals without capacity to provide informed consent (as judged by a medical clinician and household head; the household head being the listed herd or flock owner in MoA lists). Household membership was defined as either: 1) self-identifying as a household member and sleeping in the household the previous night; or 2) being a household employee. Written informed consent was obtained from all participating individuals at the time of sampling, along with parent or guardian consent for children aged 5–15 years. Institutional and national guidelines for the care of participating individuals were followed at all times.<br>There were 867 individuals included of whom 401 (46%) were female and 466 (54%) were male, median age of included individuals was 27 (IQR 15-44).
Ethics oversight	The present study was done with institutional ethical review board approvals from The Royal Veterinary College and London School of Hygiene & Tropical Medicine (both London, UK) and Jordan University of Science and Technology (JUST) (Irbid, Jordan).

Note that full information on the approval of the study protocol must also be provided in the manuscript.

Field-specific reporting

Please select the one below that is the best fit for your research. If you are not sure, read the appropriate sections before making your selection.

☒ Life sciences ☐ Behavioural & social sciences ☐ Ecological, evolutionary & environmental sciences

For a reference copy of the document with all sections, see nature.com/documents/nr-reporting-summary-flat.pdf

Life sciences study design

All studies must disclose on these points even when the disclosure is negative.

Sample size	Human sample size calculations were based on an expected seroprevalence of 4% among livestock-owning household members, a 4:1 ratio of COH members to NCOH, 80% power, with 90% confidence level, and a design effect of 1·25. Target sample size was calculated as 946 individuals: 757 COH members and 189 NCOH members. 160 COH (40 per region) and 40 NCOH (10 per region) were randomly selected for potential inclusion, based on an estimated mean household sample size of 4·7, with additional households randomly selected (80 COH, 20 per region; and 20 NCOH, five per region) as required.<br>A sample size estimate of 903 camels was used, with a total camel population size of 7,750 (MoA figures for Aqaba and Ma'an governorates), an expected seroprevalence of 12.1% (Al Majali et al) (36), a +2% precision, 95% confidence interval and design effect of 1.0. Seroprevalence estimates and their 95% CIs were calculated at the camel and herd levels, with herd-level prevalence defined according to herds with at least one seropositive individual.
Data exclusions	none
Replication	Replication is possible.
Randomization	We conducted a cross-sectional study between September 27th, 2017, and October 11th, 2018, among livestock-owning households in four sub-regions of southern Jordan (Aqaba East, Aqaba West, Ma'an East, and Ma'an West) (Figure 3). Livestock-owning households were randomly selected by a co-author (SN) using computer-generated randomisation lists (Stata, version 15.1) from a sampling frame provided by the MoA, comprising a list of all households owning one or more camels, and a list of those owning sheep or goats, within the four sub-regions. Both house and tent dwellings were eligible for inclusion.<br>All household members present at the time of sampling were invited to participate, except children younger than 5 years or individuals without capacity to provide informed consent (as judged by a medical clinician and household head; the household head being the listed herd or flock owner in MoA lists). Household membership was defined as either: 1) self-identifying as a household member and sleeping in the household the previous night; or 2) being a household employee<br><br>Camel sampling was conducted in Aqaba and Ma'an governorates of southern Jordan across two distinct periods during 2014–2015 and 2017–2018 (Figure 2). Due to the absence of an adequate sampling frame between February 9th 2014 and December 30th 2015, we conducted non-probabilistic sampling of herds belonging to camel-owing clients of a centrally located private veterinary practice (Al Quwayrah, Aqaba governorate) and camel-owing clients of local government veterinarians working in the study area. Between September 27th, 2017, and October 11th, 2018, we conducted a cross-sectional study using a sampling frame provided by the MoA comprising a list of all households owning one or more camels within the four sub-regions (Aqaba East, Aqaba West, Ma'an East, and Ma'an West), with herds being randomly selected by a co-author (SN) using computer-generated randomisation lists (Stata, version 15.1); (these camel-owning households being also eligible for inclusion in the parallel human study).
Blinding	The co-author described above was blinded to all study household data, however as this a cross-sectional population based study, other researchers, involved in the study field work, were not blinded.

Reporting for specific materials, systems and methods

We require information from authors about some types of materials, experimental systems and methods used in many studies. Here, indicate whether each material, system or method listed is relevant to your study. If you are not sure if a list item applies to your research, read the appropriate section before selecting a response.

Materials & experimental systems

n/a	Involved in the study	
☐	☐	Antibodies
☐	☐	Eukaryotic cell lines
☐	☐	Palaeontology and archaeology
☐	☒	Animals and other organisms
☐	☐	Clinical data
☐	☐	Dual use research of concern
☐	☐	Plants

Methods

n/a	Involved in the study	
☐	☐	ChIP-seq
☐	☐	Flow cytometry
☐	☐	MRI-based neuroimaging

Antibodies

Antibodies used	*Describe all antibodies used in the study; as applicable, provide supplier name, catalog number, clone name, and lot number.*
Validation	*Describe the validation of each primary antibody for the species and application, noting any validation statements on the manufacturer's website, relevant citations, antibody profiles in online databases, or data provided in the manuscript.*

Eukaryotic cell lines

Policy information about cell lines and Sex and Gender in Research

Cell line source(s)	*State the source of each cell line used and the sex of all primary cell lines and cells derived from human participants or vertebrate models.*
Authentication	*Describe the authentication procedures for each cell line used OR declare that none of the cell lines used were authenticated.*
Mycoplasma contamination	*Confirm that all cell lines tested negative for mycoplasma contamination OR describe the results of the testing for mycoplasma contamination OR declare that the cell lines were not tested for mycoplasma contamination.*
Commonly misidentified lines (See ICLAC register)	*Name any commonly misidentified cell lines used in the study and provide a rationale for their use.*

Palaeontology and Archaeology

Specimen provenance	*Provide provenance information for specimens and describe permits that were obtained for the work (including the name of the issuing authority, the date of issue, and any identifying information). Permits should encompass collection and, where applicable, export.*
Specimen deposition	*Indicate where the specimens have been deposited to permit free access by other researchers.*
Dating methods	*If new dates are provided, describe how they were obtained (e.g. collection, storage, sample pretreatment and measurement), where they were obtained (i.e. lab name), the calibration program and the protocol for quality assurance OR state that no new dates are provided.*

☐ Tick this box to confirm that the raw and calibrated dates are available in the paper or in Supplementary Information.

Ethics oversight	*Identify the organization(s) that approved or provided guidance on the study protocol, OR state that no ethical approval or guidance was required and explain why not.*

Note that full information on the approval of the study protocol must also be provided in the manuscript.

Animals and other research organisms

Policy information about studies involving animals; ARRIVE guidelines recommended for reporting animal research, and Sex and Gender in Research

Laboratory animals	*For laboratory animals, report species, strain and age OR state that the study did not involve laboratory animals.*
Wild animals	*Provide details on animals observed in or captured in the field; report species and age where possible. Describe how animals were caught and transported and what happened to captive animals after the study (if killed, explain why and describe method; if released, say where and when) OR state that the study did not involve wild animals.*
Reporting on sex	Livestock sex was observed and recorded by the sampling team, and included as an a priori variable in logistic regression analyses.
Field-collected samples	Camels were sampled in the field from domestic herds with owner consent and institutional ethical approvals. These are farmed livestock animals, maintained under normal conditions, representing 227 included herds from the south of Jordan.
Ethics oversight	Camel samling was done with institutional ethical review board approvals from the Royal Veterinary College, London School of Hygiene and Tropical Medicine and Jordan University of Science and Technology

Note that full information on the approval of the study protocol must also be provided in the manuscript.

Clinical data

Policy information about clinical studies

All manuscripts should comply with the ICMJEguidelines for publication of clinical research and a completedCONSORT checklist must be included with all submissions.

Clinical trial registration	*Provide the trial registration number from ClinicalTrials.gov or an equivalent agency.*
Study protocol	*Note where the full trial protocol can be accessed OR if not available, explain why.*
Data collection	*Describe the settings and locales of data collection, noting the time periods of recruitment and data collection.*
Outcomes	*Describe how you pre-defined primary and secondary outcome measures and how you assessed these measures.*

Dual use research of concern

Policy information about dual use research of concern

Hazards

Could the accidental, deliberate or reckless misuse of agents or technologies generated in the work, or the application of information presented in the manuscript, pose a threat to:

No	Yes	
☒	☐	Public health
☒	☐	National security
☒	☐	Crops and/or livestock
☒	☐	Ecosystems
☒	☐	Any other significant area

Experiments of concern

Does the work involve any of these experiments of concern:

No	Yes	
☒	☐	Demonstrate how to render a vaccine ineffective
☒	☐	Confer resistance to therapeutically useful antibiotics or antiviral agents
☒	☐	Enhance the virulence of a pathogen or render a nonpathogen virulent
☒	☐	Increase transmissibility of a pathogen
☒	☐	Alter the host range of a pathogen
☒	☐	Enable evasion of diagnostic/detection modalities
☒	☐	Enable the weaponization of a biological agent or toxin
☒	☐	Any other potentially harmful combination of experiments and agents

Plants

Seed stocks	*Report on the source of all seed stocks or other plant material used. If applicable, state the seed stock centre and catalogue number. If plant specimens were collected from the field, describe the collection location, date and sampling procedures.*
Novel plant genotypes	*Describe the methods by which all novel plant genotypes were produced. This includes those generated by transgenic approaches, gene editing, chemical/radiation-based mutagenesis and hybridization. For transgenic lines, describe the transformation method, the number of independent lines analyzed and the generation upon which experiments were performed. For gene-edited lines, describe the editor used, the endogenous sequence targeted for editing, the targeting guide RNA sequence (if applicable) and how the editor was applied.*
Authentication	*Describe any authentication procedures for each seed stock used or novel genotype generated. Describe any experiments used to assess the effect of a mutation and, where applicable, how potential secondary effects (e.g. second site T-DNA insertions, mosiacism, off-target gene editing) were examined.*

ChIP-seq

Data deposition

☐ Confirm that both raw and final processed data have been deposited in a public database such as GEO.

☐ Confirm that you have deposited or provided access to graph files (e.g. BED files) for the called peaks.

Data access links<br>*May remain private before publication.*	*For "Initial submission" or "Revised version" documents, provide reviewer access links. For your "Final submission" document, provide a link to the deposited data.*
Files in database submission	*Provide a list of all files available in the database submission.*
Genome browser session<br>(e.g. UCSC)	*Provide a link to an anonymized genome browser session for "Initial submission" and "Revised version" documents only, to enable peer review. Write "no longer applicable" for "Final submission" documents.*

Methodology

Replicates	*Describe the experimental replicates, specifying number, type and replicate agreement.*
Sequencing depth	*Describe the sequencing depth for each experiment, providing the total number of reads, uniquely mapped reads, length of reads and whether they were paired- or single-end.*
Antibodies	*Describe the antibodies used for the ChIP-seq experiments; as applicable, provide supplier name, catalog number, clone name, and lot number.*
Peak calling parameters	*Specify the command line program and parameters used for read mapping and peak calling, including the ChIP, control and index files used.*
Data quality	*Describe the methods used to ensure data quality in full detail, including how many peaks are at FDR 5% and above 5-fold enrichment.*
Software	*Describe the software used to collect and analyze the ChIP-seq data. For custom code that has been deposited into a community repository, provide accession details.*

Flow Cytometry

Plots

Confirm that:

☐ The axis labels state the marker and fluorochrome used (e.g. CD4-FITC).

☐ The axis scales are clearly visible. Include numbers along axes only for bottom left plot of group (a 'group' is an analysis of identical markers).

☐ All plots are contour plots with outliers or pseudocolor plots.

☐ A numerical value for number of cells or percentage (with statistics) is provided.

Methodology

Sample preparation	*Describe the sample preparation, detailing the biological source of the cells and any tissue processing steps used.*
Instrument	*Identify the instrument used for data collection, specifying make and model number.*
Software	*Describe the software used to collect and analyze the flow cytometry data. For custom code that has been deposited into a community repository, provide accession details.*
Cell population abundance	*Describe the abundance of the relevant cell populations within post-sort fractions, providing details on the purity of the samples and how it was determined.*
Gating strategy	*Describe the gating strategy used for all relevant experiments, specifying the preliminary FSC/SSC gates of the starting cell population, indicating where boundaries between "positive" and "negative" staining cell populations are defined.*

☐ Tick this box to confirm that a figure exemplifying the gating strategy is provided in the Supplementary Information.

Magnetic resonance imaging

Experimental design

Design type	*Indicate task or resting state; event-related or block design.*
Design specifications	*Specify the number of blocks, trials or experimental units per session and/or subject, and specify the length of each trial or block (if trials are blocked) and interval between trials.*
Behavioral performance measures	*State number and/or type of variables recorded (e.g. correct button press, response time) and what statistics were used to establish that the subjects were performing the task as expected (e.g. mean, range, and/or standard deviation across subjects).*

Acquisition

Imaging type(s) | *Specify: functional, structural, diffusion, perfusion.*

Field strength | *Specify in Tesla*

Sequence & imaging parameters | *Specify the pulse sequence type (gradient echo, spin echo, etc.), imaging type (EPI, spiral, etc.), field of view, matrix size, slice thickness, orientation and TE/TR/flip angle.*

Area of acquisition | *State whether a whole brain scan was used OR define the area of acquisition, describing how the region was determined.*

Diffusion MRI ☐ Used ☐ Not used

Preprocessing

Preprocessing software | *Provide detail on software version and revision number and on specific parameters (model/functions, brain extraction, segmentation, smoothing kernel size, etc.).*

Normalization | *If data were normalized/standardized, describe the approach(es): specify linear or non-linear and define image types used for transformation OR indicate that data were not normalized and explain rationale for lack of normalization.*

Normalization template | *Describe the template used for normalization/transformation, specifying subject space or group standardized space (e.g. original Talairach, MNI305, ICBM152) OR indicate that the data were not normalized.*

Noise and artifact removal | *Describe your procedure(s) for artifact and structured noise removal, specifying motion parameters, tissue signals and physiological signals (heart rate, respiration).*

Volume censoring | *Define your software and/or method and criteria for volume censoring, and state the extent of such censoring.*

Statistical modeling & inference

Model type and settings | *Specify type (mass univariate, multivariate, RSA, predictive, etc.) and describe essential details of the model at the first and second levels (e.g. fixed, random or mixed effects; drift or auto-correlation).*

Effect(s) tested | *Define precise effect in terms of the task or stimulus conditions instead of psychological concepts and indicate whether ANOVA or factorial designs were used.*

Specify type of analysis: ☐ Whole brain ☐ ROI-based ☐ Both

Statistic type for inference | *Specify voxel-wise or cluster-wise and report all relevant parameters for cluster-wise methods.*

(See Eklund et al. 2016)

Correction | *Describe the type of correction and how it is obtained for multiple comparisons (e.g. FWE, FDR, permutation or Monte Carlo).*

Models & analysis

n/a	Involved in the study
☐	☐ Functional and/or effective connectivity
☐	☐ Graph analysis
☐	☐ Multivariate modeling or predictive analysis

Functional and/or effective connectivity | *Report the measures of dependence used and the model details (e.g. Pearson correlation, partial correlation, mutual information).*

Graph analysis | *Report the dependent variable and connectivity measure, specifying weighted graph or binarized graph, subject- or group-level, and the global and/or node summaries used (e.g. clustering coefficient, efficiency, etc.).*

Multivariate modeling and predictive analysis | *Specify independent variables, features extraction and dimension reduction, model, training and evaluation metrics.*
